# Determining Possible Professionals and Respective Roles and Responsibilities for a Model Comprehensive Elder Abuse Intervention: A Delphi Consensus Survey

**DOI:** 10.1371/journal.pone.0140760

**Published:** 2015-12-02

**Authors:** Janice Du Mont, Daisy Kosa, Sheila Macdonald, Shannon Elliot, Mark Yaffe

**Affiliations:** 1 Women’s College Research Institute, Women’s College Hospital, Toronto, Ontario, Canada; 2 Dalla Lana School of Public Health Sciences, University of Toronto, Toronto, Ontario, Canada; 3 Ontario Network of Sexual Assault/Domestic Violence Treatments Centres, Toronto, Ontario, Canada; 4 Department of Family Medicine, St. Mary’s Hospital Centre, Montreal, Québec, Canada; 5 Department of Family Medicine, McGill University, Montreal, Québec, Canada; Federal University of Rio de Janeiro, BRAZIL

## Abstract

**Objective:**

We have undertaken a multi-phase, multi-method program of research to develop, implement, and evaluate a comprehensive hospital-based nurse examiner elder abuse intervention that addresses the complex functional, social, forensic, and medical needs of older women and men. In this study, we determined the importance of possible participating professionals and respective roles and responsibilities within the intervention.

**Methods:**

Using a modified Delphi methodology, recommended professionals and their associated roles and responsibilities were generated from a systematic scoping review of relevant scholarly and grey literatures. These items were reviewed, new items added for review, and rated/re-rated for their importance to the intervention on a 5-point Likert scale by an expert panel during a one day in-person meeting. Items that did not achieve consensus were subsequently re-rated in an online survey.

**Analysis:**

Those items that achieved a mean Likert rating of 4+ (rated important to very important), and an interquartile range<1 in the first or second round, and/or for which 80% of ratings were 4+ in the second round were retained for the model elder abuse intervention.

**Results:**

Twenty-two of 31 recommended professionals and 192 of 229 recommended roles and responsibilities rated were retained for our model elder abuse intervention. Retained professionals were: public guardian and trustee (mean rating = 4.88), geriatrician (4.87), police officer (4.87), GEM (geriatric emergency management) nurse (4.80), GEM social worker (4.78), community health worker (4.76), social worker/counsellor (4.74), family physician in community (4.71), paramedic (4.65), financial worker (4.59), lawyer (4.59), pharmacist (4.59), emergency physician (4.57), geriatric psychiatrist (4.33), occupational therapist (4.29), family physician in hospital (4.28), Crown prosecutor (4.24), neuropsychologist (4.24), bioethicist (4.18), caregiver advocate (4.18), victim support worker (4.18), and respite care worker (4.12).

**Conclusion:**

A large and diverse group of multidisciplinary, intersectoral collaborators was deemed necessary to address the complex needs of abused older adults, each having important roles and responsibilities to fulfill within a model comprehensive elder abuse intervention.

## Introduction

Elder abuse “refers to the violence, mistreatment or neglect that older adults living in either private residences or institutions may experience at the hands of their spouses, children, other family members, caregivers, service providers or other individuals in situations of power or trust. Elder Abuse also includes older adults abused by non-family members who are not in a position of power or trust" [1]. There are several main types of elder abuse: physical abuse (e.g., assault, threat with a weapon, restraint), sexual abuse (e.g., nonconsensual sexual contact), emotional abuse (e.g., verbal threats, humiliation), neglect (e.g., failure of a caregiver to provide adequate nutrition, hygiene, clothing, shelter, or access to necessary healthcare), and financial abuse (e.g., misuse or theft of money or possessions, use of coercion or deception to surrender finances or property) [2]. Depending on the actions being taken against the older adult, elder abuse may be a punishable crime [3].

The causes of elder abuse are multifactorial. In a recent systematic review of 49 studies, 13 common risk factors for elder abuse were identified among community dwelling older adults [4]. These factors were victim related (cognitive impairment, behavioural problems, psychiatric illness/psychological problems, functional dependency, poor physical health/frailty, low income/wealth, trauma/past abuse, ethnicity, low social support, and living with others), perpetrator related (caregiver burden/stress and psychiatric illness/psychological problems) and, more generally, family disharmony, poor, or conflictual relationships.

Although there is evidence that only a fraction of cases are detected, diagnosed, and brought to the attention of authorities [5], the abuse of older adults is known to be widespread and has significant deleterious consequences. In a 2013 review of studies globally, the prevalence of elder abuse overall ranged from 1.1% in the United States to 44.6% in Spain [6]. Rates of psychological abuse specifically have been reported from 4.6% to 27.3%; physical abuse (including sexual abuse), 0.1% to 4.9%; financial/material abuse, 2.0% to 13.6%; and neglect 5.1% to 18% [7–11]. Older women and men who have been abused may as a result experience depression, anxiety, post-traumatic stress disorder, loneliness, anger, disappointment, grief, fear, alcohol abuse, loss of property or money, physical injuries, and an increased risk of mortality, as well as rate their overall health as poorer [12].

Given the complex nature of elder abuse, the use of multidisciplinary teams is the recommended gold standard for programs, policies, and practices [13–16], as no single discipline or sector alone has the resources or expertise needed to address the issue [14, 17, 18]. However, there is currently a paucity of comprehensive health-based interventions that appropriately address elder abuse, specifically programs of care that simultaneously attend to the psychological, physical, legal, and social needs of abused older adults [19, 20]. The few existing such forensic multidisciplinary elder abuse programs have reported that processes are more streamlined (e.g., referral for medical and neuropsychological assessments is more rapid), cooperation and group problem solving among key professionals is improved (e.g., law enforcement investigators are better supported by other professionals), and the rates of prosecution for some types of elder abuse have increased [16, 21–25].

Dunlop et al. (2002) have recommended basing an elder abuse intervention on the hospital-based violence treatment centre model, which successfully “pools diverse resources” ([26], p. 114), including health, psychosocial, and medico-legal services to address the complex needs of sexual assault and intimate partner violence victims [27]. In Canada, Ontario has 35 such Sexual Assault/Domestic Violence Treatment Centres (SA/DVTCs), led by specially trained Sexual Assault Nurse Examiners (SANEs), who provide care 24/7 to women, men, and children who have been sexually assaulted and/or physically assaulted by an intimate partner and who present in an emergency department [28, 29]. However, despite the prevalence and gravity of elder abuse, within these SA/DVTCs there is no standardized program-wide provision of dedicated care for older adults who have experienced various types of abuse [20, 30].

Building on the infrastructure of Ontario’s Network of 35 SA/DVTCs, we are advancing a multi-phase, multi-method program of research to develop, implement, and evaluate a comprehensive hospital-based nurse examiner elder abuse intervention. In the first phase, in July 2012, program leaders of these centres participated in a survey to evaluate the perceived need for and feasibility of expanding their centre mandates to include a dedicated elder abuse care program. More than 80% of the 33 respondents whose centres serve adults stated that the development of an elder abuse response to all types of abuse and maltreatment should be a priority, identifying the importance of coordination of services with other key health professionals (e.g., family doctors, social workers) and community partners (e.g., public guardian and trustee) [30]. In the next phase of our research program, we conducted a systematic scoping review of elder abuse responses and identified professionals and corresponding roles and responsibilities that might be relevant to a comprehensive hospital-based intervention. In this systematic scoping review, less than 10% of the included elder abuse responses examined had been formally evaluated [20].

As a critical next step in our development of a comprehensive hospital-based elder abuse intervention, we brought together a multidisciplinary, intersectoral group of elder abuse experts to evaluate the importance of the identified professionals and their respective roles and responsibilities. Ultimately, the elder abuse intervention under development will be evaluated across Ontario and could serve as a potential model for other communities in Canada, the United States, and beyond to adopt and adapt.

## Methods

**Fig 1:**
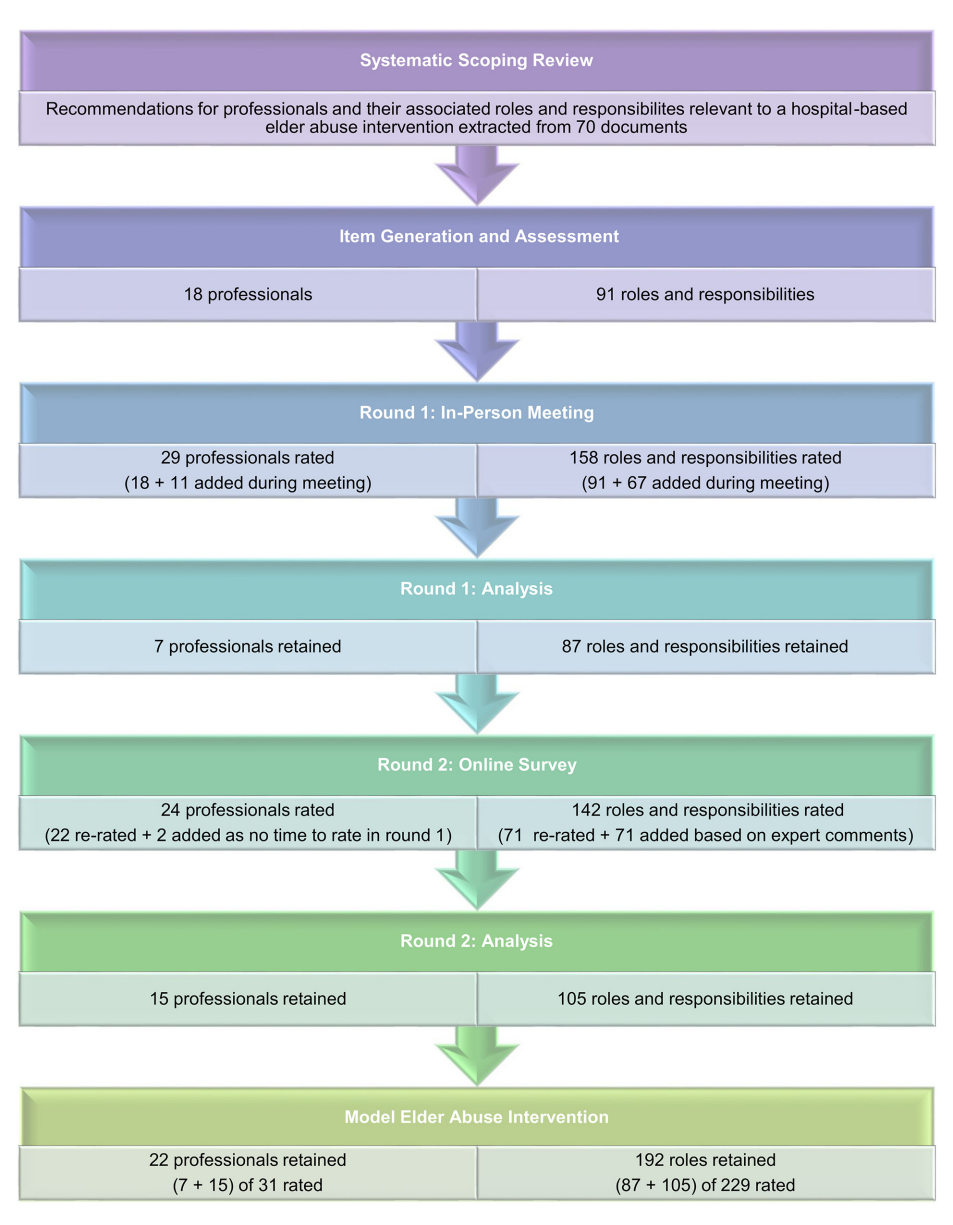
Overview of Delphi process.

To reach consensus on which professionals and associated roles and responsibilities were important to the elder abuse intervention under development, we used a modified Delphi method. This method consisted of four components: 1) an item generation process, which identified important recommendations from the literature, and preliminary assessment of the identified items, 2) formation of an expert panel that could review the recommendations, 3) an in-person meeting in which the expert panel members were presented with the list of predetermined recommendations to rate and review, and 4) an online survey in which expert panel members could re-rate items for which consensus was not achieved in the in-person meeting (see Fig 1. Overview of Delphi process). These activities were guided by current checklists and guidelines for using the Delphi method in the health literature [31, 32].

This study was approved by Women’s College Hospital Research Ethics Board (Approval Number #2013-0059-E).

### Expert panel

Multidisciplinary and intersectoral expert panel members were identified through nomination and “snowballing” based on their relevant knowledge, experience, and capacity and willingness to participate in our study [33]. We limited the number of panel members to 26 to allow for maximum interaction during the in-person meeting, of whom 21 participated in the subsequent online survey [34].

The panel represented the main stakeholder groups involved in documenting, identifying, and responding to elder abuse, setting elder abuse policy, and citizens: senior *academics* (e.g., with research expertise in elder abuse, mental health, geriatric medicine, nursing care, diversity and equity issues, and health services evaluation); *government decision-makers* (e.g., representing Employment and Social Development Canada); *healthcare providers* (e.g., nurse examiners, a social worker, a geriatrician, a family doctor, an occupational therapist); *community leaders* working in the health service, mental health, social service, finance, interfaith, emergency services, law enforcement, and legal sectors (e.g., representatives of Central Community Care Access Centre, Elder Abuse Ontario, Ombudsman Services International, Prevention of Elder Abuse Committee of York Region, Advocacy Centre for the Elderly, National Institute of Law Policy and Ageing, Metro Toronto Police Service, Public Guardian and Trustee); and *older adults* (e.g., aged 60 or older and potential consumers of health services). Many panel members represented more than one stakeholder group (e.g., lawyer also an academic expert in financial abuse and gerontology, hospital-based family physician also an elder abuse researcher). The age group, profession, workplace, and self-rated level of expertise in the elder abuse field were collected from the expert panel members.

### Item Generation and Assessment

A systematic scoping review of the scholarly and grey literature documents containing recommendations relevant to a multidisciplinary and intersectoral hospital-based elder abuse intervention was performed. This review was designed to be as inclusive as possible in order to generate a diverse perspective on the development of the intervention and to capture recommended: 1) components of care, and 2) professionals and respective roles and responsibilities. The methods and results of objective one of the systematic scoping review have been reported in detail elsewhere [20].

The extracted recommendations for the professionals and respective roles and responsibilities were assessed individually by four members of the research team (JDM, SM, DK, SE) using a clicker voting system: 1 = include, 2 = exclude, too much detail, 3 = exclude, duplicate recommendation, 4 = exclude, not compatible with forensic nurse examiner models of care, 5 = exclude, other (e.g., recommendation unclear). In order for a recommendation to be retained all 4 team members had to reach a consensus. Where there was disagreement, the recommendation was discussed until consensus was achieved. For use on the Delphi Round 1 survey, the team revised the included recommendations for consistency in terminology and style.

### Delphi Round 1

#### Survey

After item generation and assessment, there were 18 independent recommendations for professionals who could be included in the model elder abuse intervention. These professionals were categorized on the Delphi Round 1 survey as hospital-based core team: emergency physician, geriatrician, Geriatric Emergency Management (GEM) social worker, and social worker/counsellor; emergency services sector: police officer and paramedic; legal sector: Crown prosecutor, victim support worker (court), lawyer, public guardian and trustee, parole officer, and coroner; and community sector: community health worker, financial worker, dentist, caregiver advocate, spiritual advisor, and respite care worker. GEM professionals have specialized training in geriatric issues in acute care (e.g., cognitive status, safety, functional status, and self-care) so that they can rapidly assess frail, at-risk seniors and improve follow-up with appropriate services in the community [35, 36] and community health workers deliver home and community health care and connect patients to other services in the community [37]. Each professional was rated on a five point Likert scale (1 = strongly disagree, 2 = somewhat disagree, 3 = neutral, 4 = somewhat agree, 5 = strongly agree) for their importance to the comprehensive hospital-based elder abuse intervention.

After item generation and assessment, there were also 91 recommendations related to these professionals’ roles and responsibilities, which were collated for the Delphi Round 1 survey by sector: 36 recommendations for hospital-based core team (e.g., “interview the older adult”), 12 recommendations for emergency services sector (e.g., “conduct investigation of reported abuse”), 30 recommendations for the legal sector (e.g., “screen charges”), and 13 recommendations for the community sector (e.g., “recommend appropriate modifications to living situation of older adult”). Each recommendation represented an item on the survey to be rated for its importance to the intervention using the five point Likert scale.

The 109-item survey was reviewed and pre-tested by four members of the research team (JDM, SM, DK, SE) for clarity of items and reliability of the clicker voting system technology.

#### Procedure

The expert panel members were invited to a one day in-person meeting on June 16, 2014 in Toronto, Ontario. At the beginning of the meeting, the 26 member expert panel was briefed on the mandate of the Ontario Network of 35 Sexual Assault/Domestic Violence Treatment Centres (SA/DVTCs): SA/DVTC clients are seen after being medically cleared in the emergency department and offered crisis intervention, medical assessment and treatment, collection of forensic evidence, risk assessment, safety planning, follow-up medical care and counselling, and referral to various community agencies for other forms of support (e.g., shelter, legal aid, family services). For the remainder of the 8 hour meeting, the expert panel was administered the Round 1 Delphi survey in PowerPoint format.

**Fig 2:**
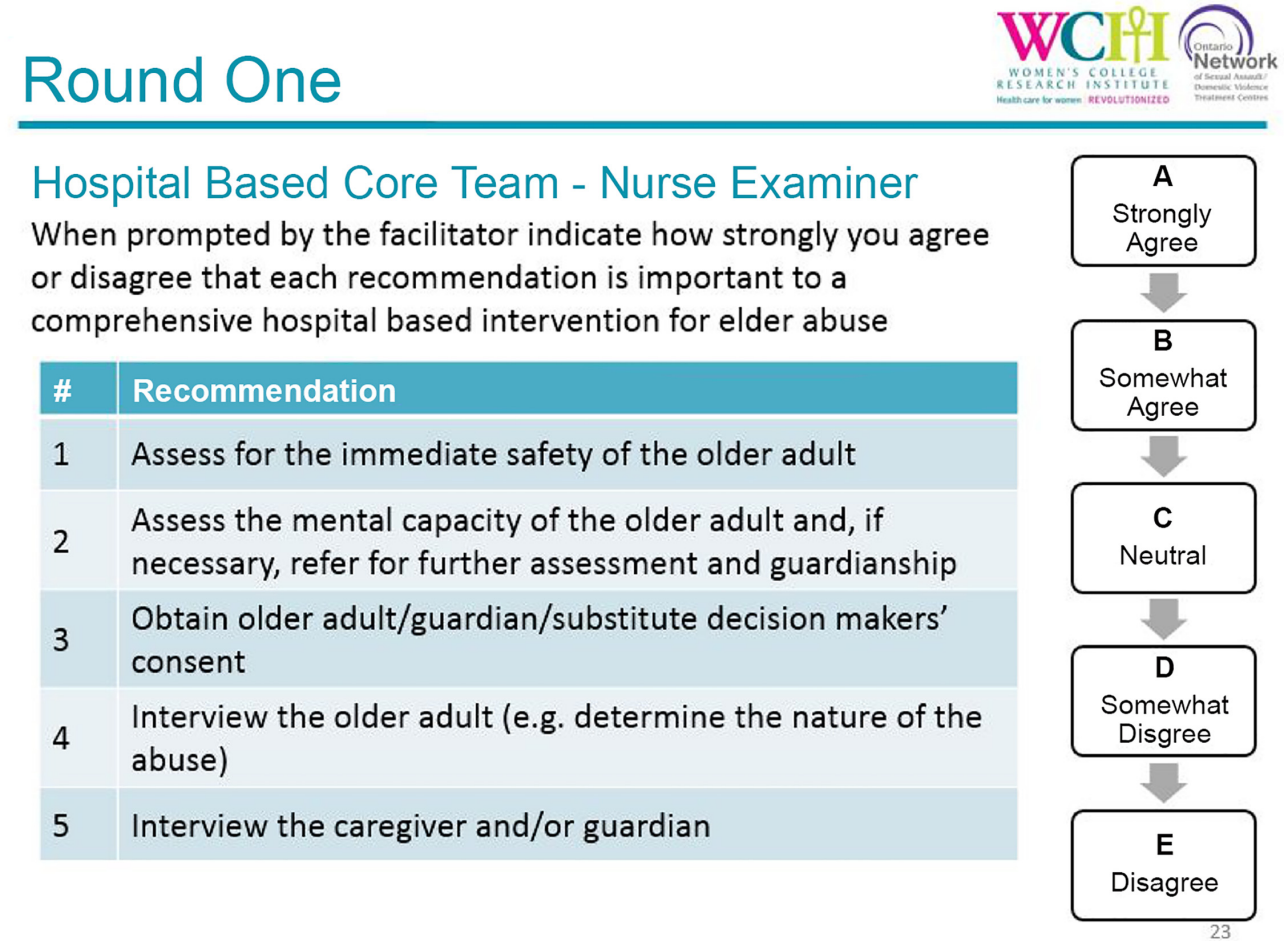
Round 1 survey slide example.

In a hybrid of nominal group and modified Delphi techniques [34, 38, 39], approximately five recommendations (items) were displayed on each slide at a time, for which there was short discussion period of up to 10 minutes moderated by the facilitators (JDM, DK), followed by a short period for individual rating of the items using a clicker voting system technology (see Fig 2. Round 1 survey slide example). All suggestions made during the discussion periods were transcribed (SE), and new recommendations were added to the Delphi Round 1 survey and subsequently rated during the meeting (11 items for professionals and 67 items for roles and responsibilities). Some of the newly identified professionals had the same or similar roles and responsibilities as some professionals already listed, but this was seen as important by the expert panel because of the variability of resources across SA/DVTC regions (e.g., availability of a geriatric psychiatrist vs geriatrician or hospital-based family physician). Based on feedback during the discussion periods, some items were reworded prior to rating.

After the discussion was terminated, and the expert elder abuse panel had rated each item in turn for importance, the group was able to see instantly the results generated if requested. Each category of professional (e.g., hospital-based core team) took between 1 and 2 hours to complete. Only the study research team had access to the individual responses and all data were later stored on a password protected and secure server, and accessed through a locked office in a secured area of Women’s College Research Institute at Women’s College Hospital.

#### Analysis

For each item rated in Round 1, a mean Likert rating and interquartile range (IQR) were calculated [32]. The IQR was used to measure the degree of consensus (IQR<1 indicates consensus achieved) for the rating for importance of each proposed item to the intervention, while the mean rating measured the average level of importance of each proposed item (mean 4+ indicates important to very important) [32]. Items with a mean Likert rating of 4+ and an IQR<1 were retained for the model elder abuse intervention. Those items with an IQR of 1+ were re-rated in Round 2.

### Delphi Round 2

#### Survey

The Round 2 survey contained 24 recommendations for possible professionals who could be included in the model elder abuse intervention, of which 22 were items that did not reach consensus in Round 1 and required re-rating, and two were items that were added for which there was no time to rate in Round 1 (shelter worker, family physician in the community). Items were re-categorized on the Delphi Round 2 survey based on expert panel feedback as hospital-based core providers including, geriatric psychiatrist, family physician in the hospital, and GEM social worker; allied healthcare collaborators including, family physician in the community, occupational therapist, physiotherapist, dietician, speech language pathologist/therapist, pharmacist, dentist, neuropsychologist, and bioethicist; legal collaborators including, public guardian and trustee, Crown prosecutor, victim support worker (court), lawyer, parole officer, and coroner; community collaborators, including financial worker, caregiver advocate, respite care worker, shelter worker, settlement worker, and spiritual advisor. The instructions for the rating of professionals were: Rate the overall importance of [specific professional]. Indicate how strongly you agree or disagree that this professional is important to a comprehensive hospital-based elder abuse intervention.

There were also 142 recommendations delineating these professionals’ roles and responsibilities included on the Round 2 survey, of which 71 were items that did not reach consensus in Round 1 and required re-rating, and 71 were items added subsequent to Round 1 based on our notes from the discussion of the expert panel. For example, the expert panel identified the need to evaluate all professionals vis-à-vis their participation on a case review team (i.e., a subset of the professionals that can be contacted regularly or as required to meet in person or by teleconference to review the details of a specific case) and, therefore, an item for the case review team was added for rating for each professional on the Round 2 survey. All recommendations were collated by professional category. The instructions for the rating of each item relating to a role or responsibility were: “For the role of [specific professional], indicate how strongly you agree or disagree that each item, to be applied where relevant, appropriate and with consent as required, is important to a comprehensive hospital-based elder abuse intervention.”

The survey was reviewed and pre-tested by five members of the research team (JDM, SM, DK, SE, MY) before being administered online for instruction and item clarity and ease of interface.

#### Procedure

In Round 2, the expert panel members were sent an email inviting them to complete the online survey within three weeks from the date of receiving the email. A unique study identification number and a link to the survey were included. Upon clicking on the survey link, the potential participant was directed to a written preamble outlining the conditions associated with study participation and a statement that completion of the survey indicated consent to participate in the study. The online survey itself was hosted on Survey Monkey, a third party website and online survey administration software (http://www.surveymonkey.com). Three reminders to complete the survey were sent at two, four, and six weeks from the initial communication.

#### Analysis

For each recommendation rated in Round 2, a mean rating and IQR were calculated. Items with a mean Likert rating of 4+ (important to very important) and an IQR<1 and/or for which 80% of ratings were 4+ were retained for the model elder abuse intervention. Eighty percent has been used as a threshold signifying a ‘strong’ level of agreement in other Delphi consensus studies [40, 41].

## Results

The expert panel of 26 represented diverse professional expertise. Categories which were non-mutually exclusive included: seven (27%) members who were identified as academics/researchers (27%), four (15%) as nurse examiners, four (15%) as elder abuse consultants, three (12%) as lawyers, three (12%) as policy-makers, three (12%) as older adults, two (8%) as financial managers, two (8%) as physicians (family physician, geriatrician), and one (4%) each as a community health manager, an occupational therapist, a pharmacist, a police officer, a public guardian and trustee manager, a retirement home and long-term care manger, and a social worker. Half (50%) of the panel members fell within the 46 to 60 years age group; only two panel members were under age 30. Approximately one quarter worked in government (27%), non-governmental organizations/civil society organizations (27%), or hospitals (23%). The remaining members worked either in a research centre (15%) or at a university (8%). Most (77%) expert panel members reported their level of elder abuse knowledge as high or in the mid to high range (see Table 1).

**Table 1:** Characteristics of expert panel members.

**Profession**	**n**	**%**
Academic/researcher	7	27%
Community health care manager	1	4%
Elder abuse consultant	4	15%
Financial manager	2	8%
Lawyer	3	12%
Nurse examiner	4	15%
Occupational therapist	1	4%
Older adult	3	12%
Pharmacist	1	4%
Physician	2	8%
Police officer	1	4%
Policy-maker	3	12%
Public guardian and trustee manager	1	4%
Retirement home and long-term care manager	1	4%
Social worker	1	4%
**Age group**	**n**	**%**
20–30	2	8%
31–45	8	31%
46–60	13	50%
61+	3	12%
**Work environment**	**n**	**%**
Government	7	27%
Hospital	6	23%
Non-governmental organization/civil society organization	7	27%
Research centre	4	15%
University	2	8%
**Level of knowledge of elder abuse**	**n**	**%**
High level	4	24%
Mid-high level	9	53%
Mid level	3	18%
Mid-low level	0	0%
Low level	1	6%

*Categories are not mutually exclusive.

### Delphi Rounds

Overall, 260 independent recommendations were rated, of which 214 (22 recommendations for professionals and 192 recommendations for roles and responsibilities) were retained for our model elder abuse intervention.

### Professionals Retained for the Model Elder Abuse Intervention

In Round 1, 29 professionals were rated, 18 from the initial generation and assessment of recommendations from the systematic scoping review and 11 added during the meeting. Seven of these professionals achieved a mean rating of 4+ (important to very important) and consensus (IQR<1). Round 2 analysis found that 15 of the 24 professionals rated (22 re-rated and 2 new items added that were generated but not rated in Round 1) achieved a mean rating of 4+ and either consensus or a high level of agreement (80% of ratings were 4+). Overall, 22 of 31 professionals rated were retained for our model elder abuse intervention.

**Fig 3:**
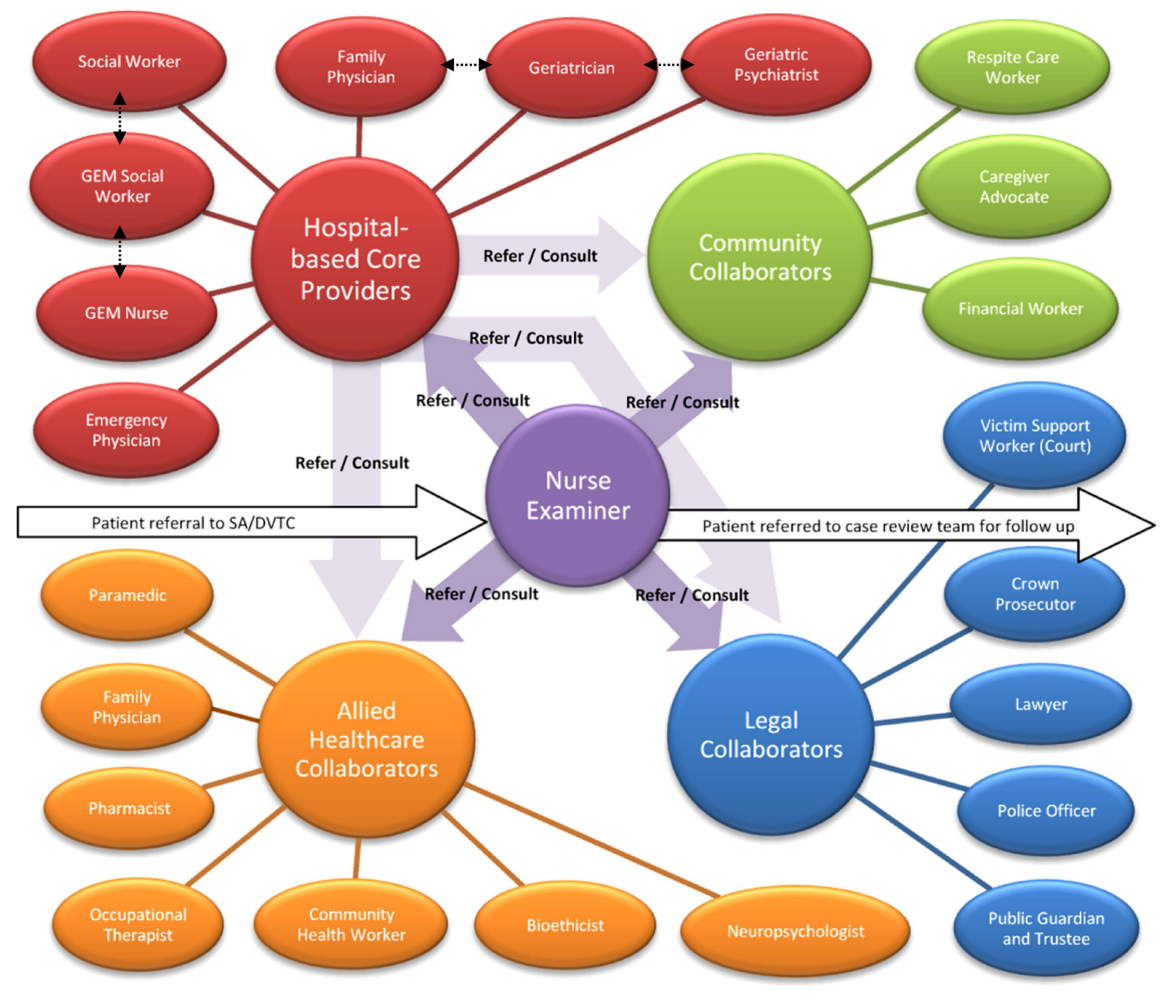
Professionals retained for the model comprehensive hospital-based elder abuse intervention. The dashed arrows represent professionals who were rated for roles and responsibilities that were sometimes the same or similar.

Professionals retained for the model elder abuse intervention were hospital-based core providers: geriatrician (mean rating = 4.87), GEM nurse (4.80), GEM social worker (4.78), social worker/counsellor (4.74), emergency physician (4.57), geriatric psychiatrist (4.33), family physician in hospital (4.28); allied healthcare collaborators: community health worker (4.76), family physician in community (4.71), paramedic (4.65), pharmacist (4.59), occupational therapist (4.29), neuropsychologist (4.24), and bioethicist (4.18); legal collaborators: public guardian and trustee (4.88), police officer (4.87), lawyer (4.59), Crown prosecutor (4.24), and victim support worker (court) (4.18); and community collaborators: financial worker (4.59), caregiver advocate (4.18), and respite care worker (4.12) (see Table 2 and Fig 3. Professionals retained for the model comprehensive hospital-based elder abuse intervention).

**Table 2:** Retained professionals for a model comprehensive hospital-based elder abuse intervention.

Professional	Mean Likert rating	Interquartile range	% Likert rating 4+	Retained items by round
**Hospital-based core providers**				
Geriatrician	4.87	0.00	100.0%	1
GEM nurse	4.80	0.00	95.2%	1
GEM social worker	4.78	0.00	94.4%	2
Social worker/counsellor	4.74	0.50	100.0%	1
Emergency physician	4.57	0.00	83.3%	1
Geriatric psychiatrist	4.33	1.00	88.9%	2
Family physician in hospital	4.28	1.00	88.9%	2
**Allied healthcare collaborators**				
Community health worker	4.76	0.00	95.5%	1
Family physician in community	4.71	0.75	100.0%	2
Paramedic	4.65	0.50	91.7%	1
Pharmacist	4.59	1.00	100.0%	2
Occupational therapist	4.29	1.00	94.1%	2
Neuropsychologist	4.24	1.00	88.2%	2
Bioethicist	4.18	1.00	82.4%	2
Dietician	4.06	1.00	76.5%	N.R.
Physiotherapist	3.94	0.00	76.5%	N.R.
Speech language pathologist/therapist	3.53	1.00	47.1%	N.R.
Dentist	3.53	1.00	52.9%	N.R.
**Legal collaborators**				
Public guardian and trustee	4.88	0.00	100.0%	2
Police officer	4.87	0.00	100.0%	1
Lawyer	4.59	1.00	94.1%	2
Crown prosecutor	4.24	1.00	82.4%	2
Victim support worker (court)	4.18	1.00	82.4%	2
Parole officer	3.41	1.00	41.2%	N.R.
Coroner	3.41	1.00	41.2%	N.R.
**Community collaborators**				
Financial worker	4.59	0.00	88.2%	2
Caregiver advocate	4.18	1.00	88.2%	2
Respite care worker	4.12	0.00	88.2%	2
Settlement worker	4.06	1.00	76.5%	N.R.
Shelter worker	3.94	2.00	70.6%	N.R.
Spiritual advisor	3.94	1.25	68.8%	N.R.

Note: GEM = Geriatric Emergency Management; N.R. = Not retained.

### Professional Roles and Responsibilities Retained for the Model Elder Abuse Intervention

In Round 1, 158 recommendations for roles and responsibilities were rated, 91 from the initial generation and assessment of recommendations from the systematic scoping review and 67 added during the meeting. Eighty-seven roles and responsibilities achieved a mean rating of 4+ (important to very important) and consensus (IQR<1). Round 2 found that 105 of the 142 roles and responsibilities rated (71 re-rated and 71 new items added that were generated but not rated in Round 1) achieved a mean rating of 4+ and either consensus or a high level of agreement (80% of ratings were 4+). Overall, 192 of the 229 roles and responsibilities rated were retained for our model elder abuse intervention.

For hospital-based core providers, 102 of 120 items delineating their roles and responsibilities were retained for the model elder abuse intervention (see Table 3): 7 of 7 items rated for the emergency department physician (e.g., “medically assess older adult and provide appropriate treatment”), 21 of 24 items rated for the nurse examiner (e.g., “assess for the immediate safety of older adult”), 8 of 12 items rated for the geriatric psychiatrist (e.g., “assess for and treat cognitive impairment of older adult”), 11 of 11 items rated for the geriatrician (e.g., “assess and address functional status of older adult”), 10 of 11 items rated for the family physician in hospital (e.g., “assess and address nutritional status of older adult”), 15 of 16 items rated for the GEM nurse (e.g., “conduct mental health assessment of older adult”), 15 of 18 items rated for the GEM social worker (e.g., “provide education and information on elder abuse to older adult”), and 15 of 21 items rated for the social worker/counsellor (e.g., “conduct psychosocial assessment of older adult (e.g., family supports, community group supports)”).

**Table 3:** Retained roles and responsibilities of professionals for a model comprehensive hospital-based elder abuse intervention.

Item	Mean Likert rating	Inter-quartile range	% Likert rating 4+	Retained items by round
**Hospital-based core providers**				
**Emergency department physician**	** **	** **	** **	** **
Medically assess older adult and provide appropriate treatment	4.91	0.00	95.7%	1
Check the capacity of older adult to consent to care	4.30	1.00	85.0%	2
Arrange hospitalization of older adult	4.70	0.50	95.8%	1
Interpret any medical findings resulting from the physical assessment of older adult conducted by nurse examiner	4.87	0.00	95.8%	1
Report suspected abuse(r) where required	4.55	0.25	85.0%	2
If abuse is highly suspected or confirmed and the suspected abuser is a health care professional, report to mandated professional body	4.45	0.25	80.0%	2
Participate on case review team	4.90	0.00	100.0%	2
**Nurse examiner**				
Assess for the immediate safety of older adult	5.00	0.00	100.0%	1
Provide for immediate basic needs (e.g., clothing, food) of older adult	4.00	2.00	70.0%	N.R.
Assess language/need for interpreter	4.80	0.00	100.0%	2
Consider special circumstances that may impact the way in which care is delivered or accepted (e.g., physical disability, Aboriginal status, immigration status)	4.85	0.00	100.0%	2
Check the capacity of older adult to consent to care	4.83	0.00	95.8%	1
Arrange for formal capacity assessment of older adult	4.05	2.00	70.0%	N.R.
Obtain older adult/substitute decision-maker's consent for care	4.95	0.00	100.0%	1
Interview older adult	5.00	0.00	100.0%	1
Interview caregiver, guardian, and/or substitute decision-maker	4.42	1.00	84.2%	2
Inform about and assist with available options to report suspected abuse to the police	4.74	0.50	100.0%	1
Conduct a physical assessment (e.g., document injuries, medical condition) of older adult	4.96	0.00	100.0%	1
Obtain toxicology samples for testing from older adult	4.82	0.00	91.3%	1
Collect biological samples (e.g., saliva, semen) for testing from older adult	4.87	0.00	95.8%	1
Provide forensic information and evidence to law enforcement	4.68	0.00	87.0%	1
Synthesize information from assessments and interviews and create a case summary	4.70	0.00	95.8%	1
Provide education and information on elder abuse to older adult	4.65	0.50	91.7%	1
Make referrals to/consult with other hospital-based core providers and allied healthcare collaborators	5.00	0.00	100.0%	1
Make referrals to/consult with intersectoral collaborators	4.87	0.00	95.7%	1
Develop safety plan for older adult	4.87	0.00	100.0%	1
Report suspected abuse(r) where required	4.65	0.00	90.0%	2
If abuse is highly suspected or confirmed and the suspected abuser is a health care professional, report to mandated professional body	4.65	0.00	90.0%	2
Maintain telephone or in-person contact to further monitor situation of older adult	3.75	2.00	65.0%	N.R.
Testify in guardianship and other legal proceedings	4.20	1.00	80.0%	2
Participate on case review team	4.45	1.00	85.0%	2
**Geriatric psychiatrist**	** **	** **	** **	** **
Assess for and treat cognitive impairment of older adult	4.65	1.00	94.1%	2
Assess functional status of older adult	3.82	2.00	64.7%	N.R.
Assess nutritional status of older adult	2.82	1.00	23.5%	N.R.
Perform formal capacity assessment of older adult	4.65	0.00	94.1%	2
Conduct mental health assessment of older adult	4.90	0.00	100.0%	1
Provide appropriate medical treatment to older adult	4.12	1.00	82.4%	2
Adjust/monitor complex medication regimes	4.59	0.00	88.2%	2
Arrange hospitalization of older adult	4.31	1.00	81.3%	2
Evaluate need and arrange for involuntary admission of older adult to hospital	4.64	0.00	90.9%	1
Report suspected abuse(r) where required	4.18	2.00	70.6%	N.R.
If abuse is highly suspected or confirmed and the suspected abuser is a health care professional, report to mandated professional body	4.18	2.00	70.6%	N.R.
Participate on case review team	4.88	0.00	100.0%	2
**Geriatrician**				** **
Assess for and treat cognitive impairment of older adult	4.87	0.00	95.8%	1
Assess and address functional status of older adult	4.65	0.50	91.7%	1
Assess and address nutritional status of older adult	4.58	1.00	100.0%	2
Interpret any medical findings resulting from the physical assessment of older adult conducted by nurse examiner	4.91	0.00	100.0%	1
Provide appropriate medical treatment to older adult	4.86	0.00	100.0%	1
Adjust/monitor complex medication regimes (e.g., in collaboration with pharmacist)	4.87	0.00	95.8%	1
Arrange hospitalization of older adult	4.68	0.00	90.9%	1
Evaluate need and arrange for involuntary admission of older adult to hospital	4.47	1.00	84.2%	2
Report suspected abuse(r) where required	4.53	0.50	89.5%	2
If abuse is highly suspected or confirmed and the suspected abuser is a healthcare professional, report to mandated professional body	4.53	0.00	84.2%	2
Participate on case review team	4.94	0.00	100.0%	2
**Family physician in hospital**				** **
Assess for and treat cognitive impairment of older adult	4.32	1.00	89.5%	2
Assess and address functional status of older adult	4.67	0.25	91.7%	1
Assess and address nutritional of older adult	4.65	0.50	95.7%	1
Interpret any medical findings resulting from the physical assessment of older adult conducted by nurse examiner	4.92	0.00	100.0%	1
Provide appropriate medical treatment to older adult	4.92	0.00	100.0%	1
Adjust/monitor complex medication regimes	4.40	1.00	80.0%	2
Arrange hospitalization of older adult	4.88	0.00	100.0%	2
Initiate process for assessment for involuntary admission of older adult to hospital	4.16	1.50	73.7%	N.R.
Report suspected abuse(r) where required	4.53	0.50	89.5%	2
If abuse is highly suspected or confirmed and the suspected abuser is a health care professional, report to mandated professional body	4.53	0.00	84.2%	2
Participate on case review team	4.75	0.00	91.7%	2
**GEM nurse**				** **
Act as case manager	4.43	1.00	92.9%	2
Arrange for formal capacity assessment of older adult	4.38	1.00	87.5%	2
Conduct mental health assessment of older adult	4.57	0.00	87.0%	1
Assess functional status of older adult	4.38	1.00	93.8%	2
Assess nutritional status of older adult	4.50	1.00	93.8%	2
Assess for medication issues	4.50	1.00	93.8%	2
Report suspected abuse(r) where required	4.56	0.00	87.5%	2
If abuse is highly suspected or confirmed and the suspected abuser is a health care professional, report to mandated professional body	4.56	0.00	87.5%	2
Provide education and information on elder abuse to older adult	4.69	0.25	93.8%	2
Assist older adult in finding alternative care providers	4.44	1.00	93.8%	2
Make referrals to/consult with other hospital-based core providers and allied healthcare collaborators	4.81	0.00	93.8%	2
Make referrals to/consult with intersectoral collaborators	4.69	0.25	93.8%	2
Assist with application for entry into community care services (e.g., residential care, transitional housing, returning home)	4.44	1.00	87.5%	2
Maintain telephone or in-person contact to further monitor situation of older adult	4.13	1.25	75.0%	N.R.
Convene case review team	4.31	1.00	87.5%	2
Participate on case review team	4.94	0.00	100.0%	2
**GEM social worker**				** **
Act as case manager	4.00	1.75	71.4%	2
Arrange for formal capacity assessment of older adult	4.68	0.00	90.9%	1
Conduct mental health assessment of older adult	3.40	1.00	46.7%	N.R.
Conduct psychosocial assessment (e.g., family supports, community group supports)	4.73	0.50	100.0%	2
Report suspected abuse(r) where required	4.52	0.00	91.7%	1
If abuse is highly suspected or confirmed and the suspected abuser is a health care professional, report to mandated professional body	4.61	0.00	91.7%	1
Provide education and information on elder abuse to older adult	4.71	0.00	92.9%	2
Assist older adult in finding alternative care providers	4.45	0.00	87.0%	1
Make referrals to/consult with other hospital-based core providers and allied healthcare collaborators	4.71	0.00	85.7%	2
Make referrals to/consult with intersectoral collaborators	4.93	0.00	100.0%	2
Assist older adult with application to obtain benefits*	3.52	3.00	62.5%	N.R.
Assist older adult to apply to criminal injuries compensation funds*	2.96	3.50	50.0%	N.R.
Assist older adult with access to financial relief resources (e.g., benefits, emergency funds, criminal injuries compensation funds)	4.87	0.00	100.0%	2
Assist older adult with application for entry into community care services (e.g., residential care, transitional housing, returning home)	4.87	0.00	100.0%	2
Maintain telephone or in-person contact to further monitor situation of older adult	4.87	0.00	100.0%	2
Convene case review team to review case	4.43	0.00	85.7%	2
Advise on ethical issues and dilemmas	4.40	1.00	80.0%	2
Participate on case review team	5.00	0.00	100.0%	2
**Social worker/counsellor**				** **
Act as case manager	3.43	2.75	50.0%	N.R.
Arrange for formal capacity assessment of older adult	4.91	0.00	100.0%	1
Conduct mental health assessment of older adult	3.53	1.50	60.0%	N.R.
Conduct psychosocial assessment of older adult (e.g., family supports, community group supports)	4.40	1.00	86.7%	2
Report suspected abuse(r) where required	4.70	0.00	95.7%	1
If abuse is highly suspected or confirmed and the suspected abuser is a health care professional, report to mandated professional body	4.67	0.25	91.7%	1
Provide education and information on elder abuse to older adult	4.60	1.00	93.3%	2
Assist older adult with application to obtain benefits*	4.78	0.00	100.0%	N.R.
Assist older adult to apply to criminal injuries compensation funds*	4.48	1.00	91.3%	N.R.
Assist older adult with access to financial relief resources (e.g., benefits, emergency funds, criminal injuries compensation funds)	4.70	0.50	95.7%	2
Assist older adult in finding alternative care providers	4.53	1.00	93.3%	2
Make referrals to/consult with other hospital-based core providers and allied healthcare collaborators	4.40	1.00	86.7%	2
Make referrals to/consult with intersectoral collaborators	4.73	0.00	93.3%	2
Assist older adult with application for entry into community care services (e.g., residential care, transitional housing, returning home)	4.67	0.50	93.3%	2
Maintain telephone or in-person contact to further monitor situation of older adult	4.67	0.50	93.3%	2
Convene case review team	4.47	1.00	80.0%	2
Provide individual and family counseling	4.77	0.00	95.7%	1
Conduct support groups	4.00	2.00	66.7%	N.R.
Accompany older adult to appropriate community resources	5.00	0.00	100.0%	1
Advise on ethical issues and dilemmas	3.80	2.00	60.0%	N.R.
Participate on case review team	4.67	0.50	93.3%	2
**Allied healthcare collaborators**				
**Paramedic**	** **	** **	** **	** **
Provide older adult pre-hospital clinical and environmental assessment and treatment in the home	4.78	0.00	95.8%	1
Transport older adult with suspected abuse to hospital emergency department with associated elder abuse intervention	4.65	0.50	91.3%	1
Conduct follow-up visits with older adult in the home	4.00	1.00	77.8%	N.R.
Participate on case review team	3.67	2.00	55.6%	N.R.
**Family physician in community**				** **
Provide relevant information on older adult's medical, psychological, and/or social history	5.00	0.00	100.0%	2
Provide information about prior suspected or confirmed incidents of elder abuse, how it was addressed, and why this approach was taken	5.00	0.00	100.0%	2
Provide information about the older adult's attitudes toward elder abuse disclosure outside of the doctor-patient encounter	4.79	0.00	100.0%	2
Provide information on risk factors for elder abuse for older adult, caregiver, and/or family members	4.93	0.00	100.0%	2
Provide information about possible negative consequences of separating older adult from his/her caregiver	4.79	0.00	92.9%	2
Provide follow-up with the older adult on a regular basis to look for signs or symptoms suggestive of abuse	4.86	0.00	100.0%	2
Participate on case review team	4.86	0.00	100.0%	2
**Community health worker**				** **
Act as case manager for community services	4.65	0.00	94.1%	2
Monitor older adult's situation through home visits, community clinics, etc.	4.75	0.00	96.0%	1
Conduct environmental assessment in the ‘home’ of older adult	4.88	0.00	100.0%	1
Recommend appropriate modifications to living situation of older adult	4.82	0.00	100.0%	2
Address functional status of older adult	4.71	0.00	92.0%	1
Follow-up on nutritional needs of older adult	4.79	0.00	96.0%	1
Assess older adult’s need for long-term care and assist with application	4.65	0.25	95.0%	1
Participate on case review team	4.76	0.00	100.0%	2
**Occupational therapist**				** **
Assess mental status of older adult	3.13	1.25	43.8%	N.R.
Assess and address functional/occupational status of older adult	4.67	0.00	91.7%	1
Refer older adult to community services	4.59	1.00	94.1%	2
Participate on case review team	4.44	1.00	100.0%	2
**Physical therapist**				** **
Assess and address functional status of older adult	4.76	0.00	94.1%	2
Refer older adult to community services	4.58	0.00	87.5%	1
Participate on case review team	4.47	1.00	82.4%	2
**Dietician**				** **
Assess and address nutritional disease status of older adult	4.83	0.00	100.0%	1
Assess ability to swallow of older adult	4.00	2.00	64.7%	N.R.
Participate on case review team	4.53	1.00	82.4%	2
**Speech language pathologist/therapist**				** **
Assess older adult’s speech and ability to swallow	4.35	1.00	88.2%	2
Participate on case review team	3.94	2.00	64.7%	N.R.
**Pharmacist**				** **
Assess for and address medication issues of older adult	4.88	0.00	100.0%	1
Participate on case review team	4.56	0.25	87.5%	2
**Dentist**				** **
Provide expert opinion to health provider on facial or dental injuries of older adult	4.83	0.00	100.0%	1
Provide older adult advice on dental care and reconstructive procedures	4.65	0.00	88.2%	2
Participate on case review team	3.88	2.00	64.7%	N.R.
**Neuropsychologist**				** **
Provide neuropsychological testing of older adult (e.g., memory, other cognitive abilities)	4.88	0.00	100.0%	2
Participate on case review team	4.59	1.00	88.2%	2
**Bioethicist**				** **
Advise on bioethical issues	4.81	0.00	93.8%	2
Participate on case review team	4.56	1.00	87.5%	2
**Legal collaborators**				
**Public guardian and trustee**	** **	** **	** **	** **
Arrange for formal capacity assessment of older adult	4.71	0.00	94.4%	1
Conduct an investigation to substantiate abuse	4.86	0.00	100.0%	1
Coordinate guardianship proceedings of older adult	4.81	0.00	93.8%	2
Act as litigation guardian or legal representative of older adult	4.86	0.00	95.7%	1
Work with older adult to address financial issues (e.g., close accounts, alert financial institutions to place an alert on accounts)	4.65	0.00	88.2%	2
Make decisions about treatment of older adult and admission to long-term care	4.82	0.00	95.7%	1
Make decisions about personal care of older adult	4.86	0.00	95.7%	1
Participate on case review team	4.71	0.00	94.1%	2
**Police officer**				
Conduct investigation of suspected abuse	4.91	0.00	95.8%	1
Identify criminal conduct	4.96	0.00	100.0%	1
Apply for and execute arrest and search warrants	4.96	0.00	100.0%	1
Lay charges against the suspected abuser	4.91	0.00	95.8%	1
Determine the forensic evidence to be submitted for analysis	4.91	0.00	95.8%	1
Protect the personal property of the older adult	4.59	1.00	88.2%	2
Assist with retrieval property of older adult	4.74	0.00	91.7%	1
Seize weapons	4.96	0.00	100.0%	1
Protect the personal safety of the older adult (e.g., confidential address program, victim relocation program, safety planning)	4.86	0.00	100.0%	1
Testify in court proceedings	4.96	0.00	100.0%	1
Support hospital-based core providers/case review team where safety is a concern	4.83	0.00	100.0%	1
Consult with hospital-based core providers/case review team on criminal justice proceedings and safety issues	4.57	0.00	90.5%	1
Participate on case review team	4.35	1.00	82.4%	2
**Crown prosecutor**				
Screen charge(s)	4.83	0.00	91.7%	1
Prosecute case	5.00	0.00	95.8%	1
Obtain court order	4.79	0.00	92.0%	1
Assist older adult in enrolling in confidential address program	4.06	1.00	76.5%	N.R.
Issue subpoena to secure witnesses and production of documents	4.78	0.00	87.5%	1
Present older adult impact information at sentencing	4.58	0.25	80.0%	1
Use information from case summary to craft sentencing recommendations for abuser	4.63	0.25	93.8%	2
File motion seeking sanction for violation of probation or failure to pay restitution	4.67	0.25	88.0%	1
Appear at parole hearing to resist abuser’s release or conditions of release	4.35	1.00	88.2%	2
Consult with hospital-based core providers/case review team on criminal justice proceedings and safety issues	4.76	0.00	100.0%	2
Participate on case review team	4.18	1.00	76.5%	N.R.
**Victim support worker (court)**				
Provide information on and assist older adult with involvement in the court system	4.83	0.00	92.0%	1
Provide court accompaniment for older adult	4.79	0.00	96.0%	1
Advocate for interests of the older adult in the court setting (e.g., accessibility)	4.88	0.00	96.0%	1
Participate on case review team	4.12	2.00	64.7%	N.R.
**Lawyer**				
Provide older adult information on financial abuse (e.g., undue influence around estate planning, wills, and trusts)	4.82	0.00	100.0%	2
Provide hospital-based core providers/case review team information to help older adult in suspected financial abuse cases (e.g., undue influence around estate planning, wills, and trusts)	4.76	0.00	100.0%	1
Provide older adult information on establishing or changing a power of attorney (transfer of legal authority)	4.82	0.00	100.0%	2
Provide hospital-based core providers/case review team information for older adult on establishing or changing a power of attorney (transfer of legal authority)	4.64	0.00	91.3%	1
Provide older adult information on changing or revoking authority of property decision-maker (e.g., unjust enrichment, fraudulent transfer of property)	4.82	0.00	100.0%	2
Provide hospital-based core providers/case review team information for older adult on changing or revoking authority of property decision-maker (e.g., unjust enrichment, fraudulent transfer of property)	4.77	0.00	95.7%	1
Provide older adult information on family matters (e.g., conflict resolution)	4.76	0.00	100.0%	2
Provide hospital-based core providers/case review team information on family matters (e.g., conflict resolution)	4.71	0.00	94.1%	2
Provide older adult information about (potential) involvement in the court system	4.91	0.00	100.0%	1
Provide hospital-based core providers/case review team information for older adult about (potential) involvement in the court system	4.47	1.00	88.2%	2
Participate on case review team	4.24	1.00	76.5%	N.R.
**Parole officer**				** **
Supervise suspected abuser on parole	4.50	0.75	78.3%	1
Monitor compliance of suspected abuser (on parole) with court orders (e.g., monitor testing for substance abuse, ensure that counseling and treatment programs are attended)	4.59	0.00	78.3%	1
Participate on case review team	3.88	2.00	64.7%	N.R.
**Coroner**				** **
Consult with the case review team on elder abuse prevention (e.g., lessons learned through retrospective reviews)	4.29	1.00	76.5%	N.R.
Respond to a suspicious death of an older adult seen by a hospital-based core provider	4.45	0.00	78.3%	1
Participate on case review team	3.47	3.00	58.8%	N.R.
**Community collaborators**				
**Financial worker**				** **
Provide older adult information on steps to address concerns related to assets and other material resources (e.g., close current account and open a new account that is inaccessible to others, identify suspicious transactions)	4.82	0.00	94.1%	2
Provide hospital-based core providers/case review team information on steps to address concerns of older adult related to assets and other material resources (e.g., close current account and open a new account that is inaccessible to others, identify suspicious transactions)	4.88	0.00	92.0%	1
Provide older adult information to help manage financial affairs and access the appropriate services to address problems	4.96	0.00	96.0%	1
Provide hospital-based core providers/case review team information to help the older adult manage financial affairs and access the appropriate services to address problems	4.35	1.00	82.4%	2
Participate on case review team	3.82	2.00	64.7%	N.R.
**Caregiver advocate**				
Provide education and information to caregivers (e.g., elder abuse, older adult care, resources)	4.94	0.00	100.0%	2
Provide support for caregivers	5.00	0.00	100.0%	2
Participate on case review team	4.12	2.00	70.6%	N.R.
**Respite care worker**				** **
Provide temporary shelter for older adult	4.79	0.00	100.0%	1
Participate on case review team	4.12	2.00	64.7%	N.R.
**Shelter worker**				** **
Provide temporary shelter for older female adult with suspected abuse	4.41	0.00	82.4%	2
Participate on case review team	3.59	2.00	47.1%	N.R.
**Settlement worker**				** **
Provide information and support to older adult who is an immigrant regardless of legal status (e.g., immigration and sponsorship issues, navigation of services, court accompaniment)	4.94	0.00	100.0%	2
Participate on case review team	4.12	2.00	70.6%	N.R.
**Spiritual advisor**				** **
Provide spiritual support for older adult and family	4.50	1.00	87.5%	1
Participate on case review team	3.88	2.00	64.7%	N.R.

Note: Each item is to be applied where relevant, appropriate, and with consent as required; N.R. = Not Retained; GEM = geriatric emergency management.

*Item was rated in Round 1, and in Round 2, it was collapsed with another similar item and rated as, “Assist older adult with access to financial relief resources.”

For allied healthcare collaborators, 34 out of 40 items delineating their roles and responsibilities were retained for the model elder abuse intervention (see Table 3): 2 of 4 items rated for the paramedic (e.g., “provide older adult pre-hospital clinical and environmental assessment and treatment in the home”), 7 of 7 items rated for the family physician in the community (e.g., “provide relevant information on older adult's medical, psychological, and/or social history”), 8 of 8 items rated for the community health worker (e.g., “conduct environmental assessment in the ‘home’ of older adult”), 3 of 4 items rated for the occupational therapist (e.g., “assess and address functional/occupational status of older adult”), 3 of 3 items rated for the physical therapist (e.g., “refer older adult to community services”), 2 of 3 items rated for the dietician (e.g., “assess and address nutritional disease status of older adult”), 1 of 2 items rated for the speech language pathologist/therapist (e.g., “assess older adult’s speech and ability to swallow”), 2 of 2 items rated for the pharmacist (e.g., “assess for and address medication issues of older adult”), 2 of 3 items rated for the dentist (e.g., “provide older adult advice on dental care and reconstructive procedures”), 2 of 2 items rated for the neuropsychologist (e.g., “provide neuropsychological testing of older adult (e.g., memory, other cognitive abilities)”), and 2 of 2 items rated for the bioethicist (e.g., “advise on bioethical issues”).

For legal collaborators, 46 of 53 items delineating their roles and responsibilities were retained for the model elder abuse intervention (see Table 3): 8 of 8 items rated for the public guardian and trustee (e.g., “act as litigation guardian or legal representative of older adult”), 13 of 13 items rated for the police officer (e.g., “conduct investigation of suspected abuse”), 9 of 11 items rated for the Crown prosecutor (e.g., “prosecute case”), 3 of 4 items rated for the victim support worker (e.g., “provide court accompaniment for older adult”), 10 of 11 items rated for the lawyer (e.g., “provide older adult information on establishing or changing a power of attorney (transfer of legal authority)”), 2 of 3 items rated for the parole officer (e.g., “monitor compliance of suspected abuser (on parole) with court orders (e.g., monitor testing for substance abuse, ensure that counseling and treatment programs are attended)”), and 1 of 3 items rated for the coroner (e.g., “respond to a suspicious death of an older adult seen by a hospital-based core provider”).

For community collaborators, 10 of 16 items delineating their roles and responsibilities were retained for the model elder abuse intervention (see Table 3): 4 of 5 items rated for the financial worker (e.g., “provide older adult information on steps to address concerns related to assets and other material resources (e.g., close current account and open a new account that is inaccessible to others, identify suspicious transactions)”), 2 of 3 items rated for the caregiver advocate (e.g., “provide education and information to caregivers (e.g., elder abuse, older adult care, resources)”), 1 of 2 items rated for the respite care worker (e.g., “provide temporary shelter for older adult”), 1 of 2 items rated for the shelter worker (e.g., “provide temporary shelter for older female adult with suspected abuse”), 1 of 2 items rated for the settlement worker (e.g., “provide information and support to older adult who is an immigrant regardless of legal status (e.g., immigration and sponsorship issues, navigation of services, court accompaniment)”), and 1 of 2 items rated for the spiritual advisor (e.g., “provide spiritual support for older adult and family”).

## Discussion

In a commentary in *JAMA* (*The Journal of the American Medical Association*), two leading elder abuse researchers indicated that to develop effective elder abuse interventions there is a need for research that is “innovative, multidisciplinary, [and] collaborative” [42] (p. 2461). The development, implementation, and evaluation of evidence-informed elder abuse interventions also has been identified as a priority by the Centers for Disease Control and Prevention [4, 43]. Due to the complexity of elder abuse, it is impossible and singularly minded to attempt to address the problem through any one professional lens, as no single professional group has all the necessary resources and expertise [44]. For this reason, we have undertaken a program of research to build a multidisciplinary, intersectoral elder abuse intervention.

To integrate multiple perspectives successfully into a comprehensive hospital-based nurse examiner elder abuse intervention, the roles of participating professionals must be clearly delineated to ensure that the care is integrated and avoids duplication of efforts [18, 45–47]. However, there are still considerable gaps and lack of agreement in the literature around which professionals should participate in multidisciplinary elder abuse care programs and what their responsibilities should be. In a recent review of multidisciplinary team legislative language associated with elder abuse investigations in the United States, researchers found that only a few states listed which professionals should be included in the response and none listed their specific duties, information the authors argued critical to guide elder abuse program implementation [48].

Our study helps to address these gaps in elder abuse programs and policies [20, 30]. Using a modified Delphi consensus survey method [49], we have rigorously and clearly delineated the roles and responsibilities of professionals who could comprise a comprehensive hospital-based elder abuse response. The Delphi process was utilized as it is particularly useful where high quality evidence in a specific area is lacking [31, 32, 50]. The first Delphi round was conducted in-person using a hybrid of nominal group techniques and a survey to facilitate the rapid exchange of ideas. Discussion periods prior to rating items resulted in the rewording and adding of items both during and immediately after the first round. This iterative process maximized the benefits of the Delphi method to capture opinion data from the diverse group of professionals on our expert panel. In order to facilitate efficient follow-up with the expert panel, round two was subsequently held online [40, 41].

As recommended by Tetzlaff et al. [51], great care was taken to ensure that our expert panel was comprised of representative stakeholders key to the development of a comprehensive hospital-based intervention. The panel membership was of particular importance as collectively the expertise of participants needed to span the list of professionals and their roles and responsibilities identified during item generation and assessment. By contacting potential participants using a personalized email and a follow-up phone call where required, we were successful in recruiting leading experts with predominantly a mid-high to high level of knowledge of elder abuse. The resultant diverse composition and significant expertise upholds the internal and external validity of the results of this study.

Consistent with recent recommendations for responding to elder abuse [52], in our study, a large and diverse group of multidisciplinary and intersectoral collaborators was deemed necessary to address the complex needs of abused older adults, each professional having important roles and responsibilities to fulfill within a comprehensive hospital-based elder abuse intervention. Of the 31 professionals rated for importance to the intervention, 22 were retained for our model elder abuse intervention. Although the list of professionals indicated important by our experts may seem large, some of the few already established multidisciplinary elder abuse teams are comprised of a similar range of professionals, and have reported increased referrals and streamlined processes [18, 21, 22, 24, 25, 53–55]. Additionally, in our study, certain professionals were rated for the same or similar roles and responsibilities and could replace each other in the intervention as required by local staffing and resources (e.g., GEM social worker vs social worker/counsellor).

Nine professionals—physiotherapist, speech language pathologist/therapist, dentist, dietician, parole officer, coroner, settlement worker, shelter worker, and spiritual advisor—were not retained for our model elder abuse intervention and would not be part of a formal response depicted in Fig 3, potentially requiring elder abuse intervention training and inclusion in an intersectoral agreement. However, each of these professionals had roles and responsibilities that were agreed to be important to the elder abuse intervention and, therefore, may need to be consulted on a case by case basis when their expertise is required. For example, on an *ad hoc* basis, it may be warranted to contact the coroner to respond to the suspicious death of an older adult seen by a hospital-based core provider. Overall, our findings nonetheless indicated a fairly high degree of agreement on who should be engaged and in what capacities in a comprehensive hospital-based elder abuse intervention, with the expert panel rating the majority of items as important or very important.

### Limitations

There is no standard or agreed upon framework for valid Delphi facilitation including a stopping policy for Delphi rounds. Certain biases are inherent, however, in the Delphi process that could skew the results toward consensus or dissent. For example, if members of the expert elder abuse panel drop out of the study before it is completed because they feels that the rest of the group does not share their opinions, this could result in an over-estimation of how much panel members agreed on the items [50]. However, we had an 81% retention rate of panel members between rounds and feel, therefore, that the risk of this type of bias is fairly low [56, 57]. In another example, experts may interpret the meaning of the items differently, causing polarization in the data and leading to potentially higher dissent [51]. In our study, we mitigated this source of bias by pre-testing the surveys and conducting the first round of the Delphi in-person, where each item could be discussed before voting to clarify its context, wording, and content.

## Conclusion and Future Steps

There are very few rigorously designed studies that have developed and evaluated comprehensive elder abuse interventions [19, 20, 55]. Results from the Delphi consensus survey presented in this study begin to address this paucity of evidence, as key roles and responsibilities of professionals who could comprise a comprehensive hospital-based nurse examiner elder abuse intervention have been established. In a critical next step, we will use this information to guide the development of a template protocol to facilitate cooperation and streamline processes between intersectoral collaborators and SA/DVTC sites [47, 58]. Although the elder abuse intervention being developed is for implementation and evaluation in Ontario, Canada, the findings of this study are of potential utility to other jurisdictions, as nurse examiner led sexual assault services that could provide the infrastructure for a hospital-based elder abuse response are increasingly being established globally [27]. Also, our methodology could be used by others to perform a similar analysis of what is needed locally for an effective comprehensive elder abuse response.
